# ^18^F-florbetaben whole-body PET/MRI for evaluation of systemic amyloid deposition

**DOI:** 10.1186/s13550-018-0425-1

**Published:** 2018-07-24

**Authors:** Lucia Baratto, Sonya Youngju Park, Negin Hatami, Praveen Gulaka, Shreyas Vasanawala, Thomas Koshy Yohannan, Robert Herfkens, Ronald Witteles, Andrei Iagaru

**Affiliations:** 1 Division of Nuclear Medicine and Molecular Imaging, Stanford University Medical Center, 300 Pasteur Dr, Room H-2200, Stanford, CA 94305 USA; 2 Department of Radiology, Stanford University, Stanford, CA 94305 USA; 3 Department of Medicine, Stanford University, Stanford, USA

**Keywords:** ^18^F-florbetaben, PET/MR, Amyloid, Cardiac, Systemic

## Abstract

**Background:**

Florbetaben, a ^18^F-labeled stilbene derivative (Neuraceq®, formerly known as BAY-949172), is a diagnostic radiopharmaceutical developed to visualize β-amyloid plaques in the brain. Here, we report a pilot study evaluating patients with suspected cardiac amyloidosis for systemic extent of disease.

**Methods:**

We prospectively enrolled nine patients, 61–86 year old (mean ± SD 69.4 ± 8.6), referred from the cardiac amyloid clinic. First, dynamic imaging of the heart was acquired immediately after injection of 222–318.2 MBq (mean ± SD 270.1 ± 33.3) of ^18^F-florbetaben using the GE SIGNA PET/MRI. This was followed by a whole-body PET/MRI scan 60–146.4 min (mean ± SD 98 ± 33.4) after injection. Cardiac MRI sequences included ECG-triggered cine SSFP, T2-weighted, and late gadolinium-enhanced imaging. Whole-body MRI sequences included MRAC and axial T1-weighted imaging.

**Results:**

High early uptake and delayed high uptake in the left ventricle correlated with amyloid deposition in five patients, while low uptake on early and delayed cardiac imaging was noted in four patients. Cardiac function measurements were successfully obtained in all participants. Areas of increased abnormal ^18^F-florbetaben accumulation were noted on delayed whole-body imaging in the bone marrow (seven patients), stomach (diffuse in five patients and focal in one patient), brain (five patients), salivary glands (three patients), tongue (three patients), spleen (three patients), skeletal muscles (three patients), ocular muscles (two patients), thyroid (two patients), pleura (two patients), kidneys (two patients), and lungs (two patients).

**Conclusions:**

Whole-body ^18^F-florbetaben PET/MRI is promising for localization of systemic amyloid deposition. This technique may provide important structural and functional information regarding the organs involved by disease, with potential to guide biopsy and evaluate response to treatment.

**Trial registration:**

Clinicaltrials.gov registration: NCT03119558.

**Electronic supplementary material:**

The online version of this article (10.1186/s13550-018-0425-1) contains supplementary material, which is available to authorized users.

## Introduction

Amyloidosis is a heterogeneous group of disorders characterized by the extracellular deposition of insoluble proteins in a beta-pleated sheet conformation [1]. Systemic deposition of these amyloid fibrils results in organ damage and dysfunction.

Cardiac amyloidosis, which is usually related to deposition of an improperly folded light chain protein (AL) or transthyretin [2], is often not diagnosed until late in the course of disease, when patients present with heart failure. The amyloid fibrils get the heart increasingly stiff, impairing the left ventricular (LV) function [1]. As cardiac amyloidosis confers a significantly worse long-term prognosis, it is very important to diagnose the disease before clinical manifestation of heart failure because it may permit early initiation of appropriate therapy. Currently, diagnosis requires many tests, including physical examination, cardiac troponin, B-type natriuretic peptide, ECG, echocardiogram, and cardiac MRI [3]. The diagnostic challenge lies in differentiating amyloidosis from other causes of LV hypertrophy. If the disease is systemic, a biopsy of other involved organs is often used to confirm the diagnosis, but when the patient is persistently symptomatic but the diagnosis of amyloidosis cannot be confirmed, a myocardial biopsy is necessary.

^18^F-florbetaben specifically binds to the amyloid β-pleated-sheet structure and PET imaging with this radiotracer has been shown to accurately detected β-amyloid neuritic plaques in the brain in patients with Alzheimer disease [4]. Based on these results, ^18^F-florbetaben has also been evaluated for cardiac amyloidosis [5–7] and found it was able to accurately identify and differentiate between cardiac amyloidosis (both Al and TTR) and hypertensive heart disease [5].

Here, we investigated the potential role of ^18^F-florbetaben PET/MRI imaging in the diagnosis of cardiac amyloidosis. Moreover, given the potential of amyloidosis to be a systemic disease, we performed a whole-body scan to determine other sites of involvement. In this brief communication, we present the first nine patients we studied using ^18^F-florbetaben PET/MRI.

## Materials and methods

### Study population

Consecutive patients ≥ 18 year old with known or suspected cardiac amyloidosis were prospectively recruited from the cardiac amyloid clinic for study enrollment from May 2016 to April 2018. Participants were excluded if they were pregnant or nursing, or if they had contraindications for MRI (e.g., metallic implants). The local institutional review board (IRB) approved this study and written consent was obtained from all patients before participation in the study. The study is compliant with Health Insurance Portability and Accountability Act (HIPAA) guidelines.

### Imaging acquisition

Dynamic PET imaging of the heart started immediately after injection of a mean dose of 222–318.2 MBq (mean ± SD 270.1 ± 33.3) of ^18^F-florbetaben. This was followed by whole-body PET/MRI image acquisition at 60–146.4 min (mean ± SD 98 ± 33.4) post-injection with a scan duration of 80.9 ± 38.9 min (means ± SD), range 20–120 min.

PET/MR examinations were performed with a whole-body imaging system capable of simultaneous PET and MR Imaging (SIGNA, GE Healthcare, Milwaukee, WI). The system consists of a 3T MR imager that contains a PET detector based on lutetium oxyorthosilicate scintillators coupled to silicon photomultiplier detector (SiPM) technology. PET events were detected throughout the entire cardiac MRI acquisition in one bed position centered over the heart. A coronal T1-weighted 3D encoded spoiled gradient-echo sequence was acquired for attenuation correction.

More details of the image acquisition are shown in Additional file 1.

### Image analysis

Studies were analyzed blinded to all clinical information. PET image analysis was performed using dedicated viewing and post-processing software (Advantage Workstation, GE Healthcare, WI, USA). Cardiac and whole-body PET images were interpreted by three nuclear medicine physicians (SP, LB, AI) in consensus.

For cardiac MRI analysis, left and right ventricular (LV and RV) endocardial borders were manually contoured on short-axis steady-state free precession (SSFP) images to assess for end-diastolic and end-systolic volumes, and ejection fraction (EF).

Cardiac amyloidosis was confirmed when early and delayed ^18^F Florbetaben uptake was noted, as previously reported [5, 8].

Areas of increased tracer accumulation were recognized on the delayed images and the maximum standardized uptake value (SUV_max_) was collected, using a volume of interest (VOI) of variable size.

## Results

All nine patients who were enrolled had no history of prior treatment for amyloidosis. Seven patients had biopsy-proven systemic amyloidosis at the inclusion in the study, including five with known cardiac involvement. Two patients were referred to differentiate between hypertrophic cardiomyopathy and amyloidosis. Table 1 shows clinical characteristics of the enrolled participants. Cardiac function measurements were successfully obtained in all participants.

**Table 1:** Clinical and laboratory data from participants included in the study

Characteristics	Patients								
	1	2	3	4	5	6	7	8	9
Age	61	66	70	80	72	86	62	66	62
Sex	F	M	M	M	M	M	M	M	M
Diagnosis	AL Lambda	AL Lambda	AL Kappa	HCM	AL Lambda	HCM	AL Lambda	AL Lambda	AL Lambda
Biopsy	Abdominal Fat^a^	Tongue Lungs BM^b^	Kidneys	–	Myocardial BM^a^	–	Abdominal fat BM^b^	GI BM^b^	Kidneys
Organs involvement^a^	Heart Kidney Larynx/GI	Heart Kidneys	–	–	–	–	Heart	Kidneys	Heart
Troponin I (< 0.1 ng/ml)	< 0.1	< 0.1	< 0.1	< 0.1	1.986	< 0.1	< 0.01	< 0.1	0.08
NT-proBNP (< 300 mg/ml)	2.086	8.804	631	3.188	1.804	N/A	330	183	575
Creatinine (0.50–1.2 mg/dl)	0.49	1.6	1.76	1.45	1.66	1.03	0.7	1.8	2.61
Alb/creat ratio (< 30 mg/g)	2.214	1.972	54	N/A	N/A	N/A	N/A	N/A	N/A
Free Lambda light chain (0.6–2.6 mg/dl)	3.1	16.7	1	N/A	0.3	N/A	2.7	4.3	8.4
Free Kappa light chain (0.3–2.0 mg/dl)	1.5	1.5	1.7	N/A	0.1	N/A	0.4	3.5	9.2
Free K/L ratio (0.3–1.6)	0.4	0.1	1.7	N/A	0.3	N/A	0.1	0.8	1.1

*N/A* not available

^a^Organs involvement was diagnosed by patient 1: myocardium (echo), kidneys (laboratory), laryngeal/GI (clinically); patient 2: myocardium (cardiac MRI), kidneys (laboratory); patient 7: myocardium (echo); and patient 8: kidneys (laboratory)

^b^BM biopsy was performed and was positive for plasma cells, but negative for Congo-red stain

High early uptake and delayed high uptake in the left ventricle correlated with amyloid deposition in five patients, while low uptake on early and delayed cardiac imaging was noted in four patients. Imaging characteristics of patients with myocardial amyloidosis are shown in Table 2. Functional measurements from echocardiography and cardiac MRI (from the dynamic PET/MRI scan) are shown in Table 3.

**Table 2:** Imaging characteristics of patients with myocardial amyloidosis

	Patient 1	Patient 2	Patient 5^a^	Patient 7	Patient 9
Echocardiography	Moderate concentric LVH with echo-texture suspicious for amyloid	Moderate concentric LVH	Moderate concentric LVH	Moderate concentric LVH with echo-texture suspicious for amyloid	Moderate LVH with echo-texture suspicious for amyloid
Cardiac MRI	N/A	BH with extensive sub-endocardial delayed enhancement consistent with CA	N/A	N/A	N/A
^18^F-florbetaben PET/MRI	Elevated early and delayed uptake in LV	Elevated early and delayed uptake in LV	Elevated early and delayed uptake in LV	Elevated early and delayed uptake in LV	Elevated early and delayed uptake in LV

*LVH* left ventricular hypertrophy, *BH* biventricular hypertrophy, *CA* cardiac amyloidosis, *LV* left ventricle, *N/A* not available

^a^Myocardial involvement was proven by biopsy

**Table 3:** Echocardiographic and MRI functional measurements

Echo vs MRI parameters	Patient								
	1	2	3	4	5	6	7	8	9
LV ejection fraction (%)									
Echo	51.4	40.6	67.0	61.0	39.6	65	54.6	51.9	35.0
MRI	63.0	38.0	50.0	60.0	35.0	59.0	44.0	52.0	26.0
End-diastolic volume (ml)									
Echo	77.4	47.7	66.1	58.0	139.9	N/A	N/A	122.7	119.5
MRI	94.1	86.6	100.0	84.7	140.0	64.6	152.0	132.0	164.0
End-systolic volume (ml)									
Echo	37.6	28.3	21.8	22.6	84.5	N/A	N/A	59.1	77.3
MRI	34.9	53.5	50.1	33.6	90.6	26.5	84.4	64.0	122.0
Time between echocardiogram and ^18^F-florbetaben PET/MRI	3 d	1 m	3 m	5 d	2 m	6 m	1.5 m	1 m	6 m

*N/A* not available, *d* days, *m* months

All patients had moderate ^18^F-florbetaben uptake in the liver and gallbladder in keeping with hepatobiliary excretion of ^18^F-florbetaben.

We considered the uptake to be physiological if it had at least one of the following characteristics: low grade (SUV_max_ < 2.5), symmetrical for paired organs and already described as physiological in the existing literature [9, 10]. Areas of increased abnormal ^18^F-florbetaben accumulation were noted on delayed whole-body imaging in the bone marrow (seven patients), stomach (diffuse in five patients and focal in one patient), brain (five patients), salivary glands (three patients), tongue (three patients), spleen (three patients), skeletal muscles (three patients), ocular muscles (two patients), thyroid (two patients), pleura (two patients), kidneys (two patients), and lungs (two patients).

**Fig. 1:**
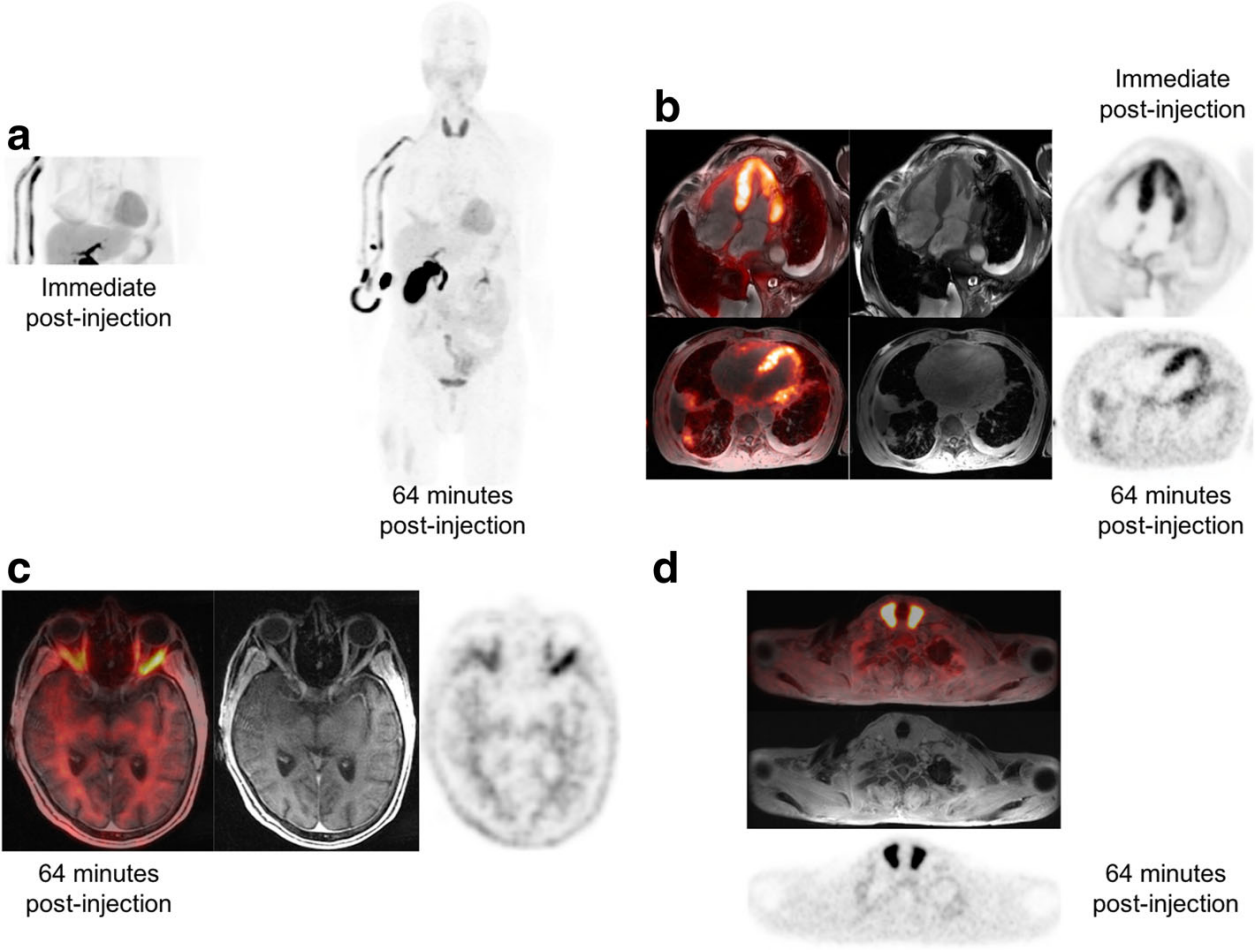
**a** MIP and **b** cardiac bed images from participant #2 show intense myocardial ^18^F-florbetaben uptake, suspicious for amyloid. Intense uptake was also demonstrated in the extraocular muscles, consistent with recent orbital MRI findings that were concerning for amyloid involvement (**c**). Although such manifestation is rare, intense uptake was noted in the thyroid glands (**d**). The patient had mild or subclinical hypothyroidism, with low free thyroxine (1.1μg/dl) and upper limit of the TSH normal range (3.85–4.6 mIU/L). Physical examination of the neck also revealed discomfort on palpation

**Fig. 2:**
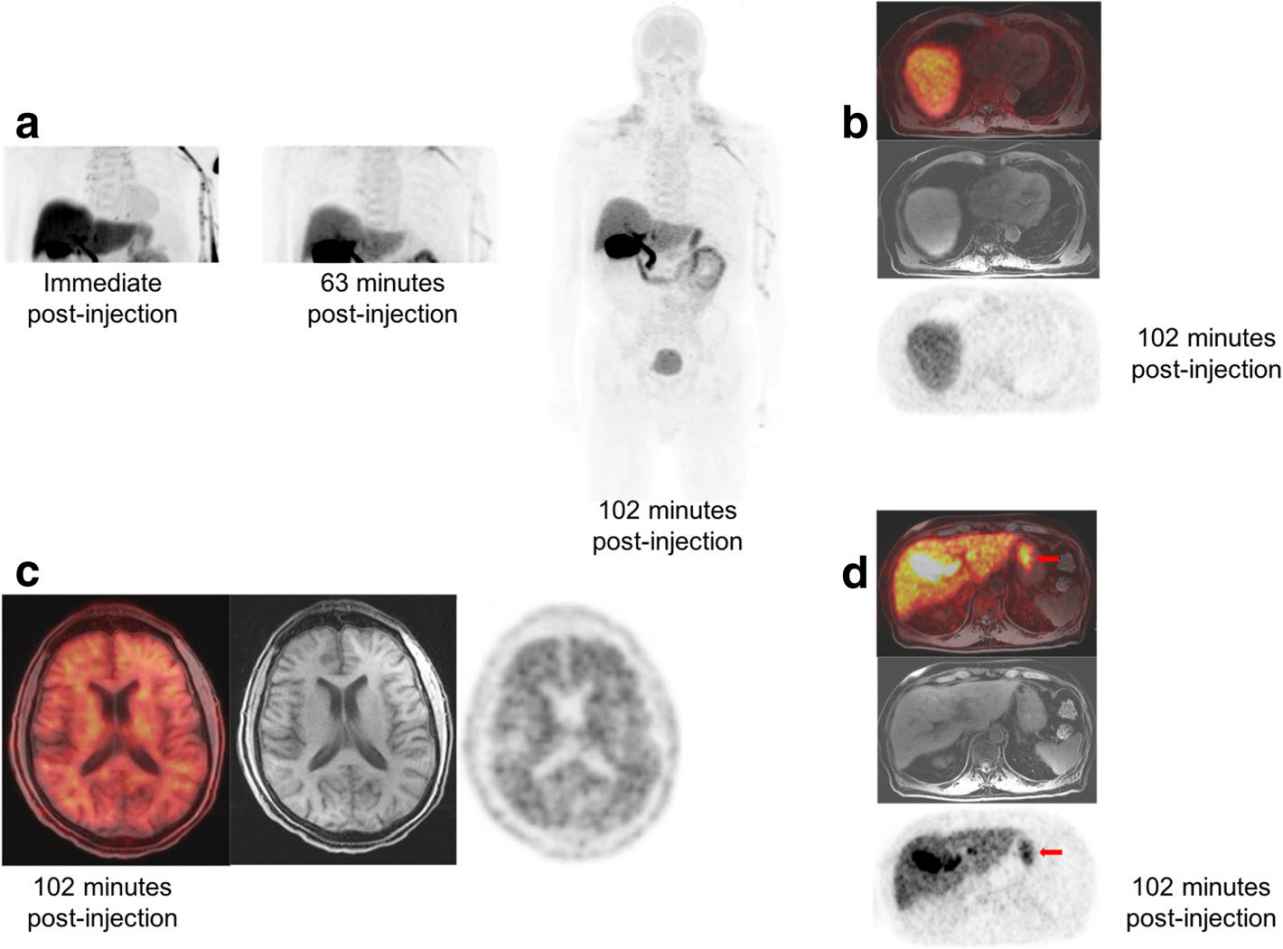
**a** MIP and **b** cardiac bed images from participant #3 with known renal and bone marrow involvement show very low myocardial ^18^F-florbetaben uptake, but diffuse uptake in the stomach (**c**). Another interesting finding is the diffuse uptake in the gray matter with loss of gray-white matter separation (**d**). Given the patient’s age of 70 years, further neurological evaluation was recommended

Systemic ^18^F-florbetaben uptake and corresponding MRI findings are shown in Table 4. Illustrative images from two cases are shown in Figs. 1 and 2.

**Table 4:** SUV_max_ measurements in extracardiac sites of increased ^18^F-florbetaben uptake on delayed images

Site of uptake	Patients									SUV_max_ (mean)	Patients with uptake (%)	Clinical/pathological organs involvement
	1	2	3	4	5	6	7	8	9			
Bone marrow	3.2^a,b^	–	2.4^a^	3.2^a^,^b^	3^a^	3.4^a^	–	3.4^a,b^	4.7^a^	3.3	7/9 (77%)	4/9 (44%)
Spleen	2.6^a^ (D)	–	1.1 (D)	–	1.2 (D)	1.1 (D)	–	25.1^a^ (D)	16.7^a^ (D)	8	6/9 (66%)	
Kidneys	1.6	1.8	1.3	1.9	2.1	1.9	1.9	6.4^a^	5.9^a^	2.8	9/9 (100%)	5/9 (55%)
Stomach	3.7^a^ (D)	3.9^a^ (D)	11.9^a,b^ (F)	4.9^a^ (D)	31.2^a^ (D)	5.9^a^ (D)	–	–	–	10.3	6/9 (66%)	2/9 (22%)
Parotids	1.2 (B)	4 (B)	3.4 (B)	9.4^a^ (B)	4.5^a^ (B)	3.5 (B)	1.1 (B)	2.4 (B)	5.3^a^ (B)	3.9	9/9 (100%)	
Submandibular Glands	–	–	–	–	–	–	–	2.9 (B)	4.4^a^ (B)	3.7	2/9 (22%)	
Lungs	–	2.2^a,b^ (F)	–	–	–	–	2.7^a^ (D)	–	–	2.5	2/9 (22%)	1/9 (11%)
Thyroid	–	11.5^a^ (B)	–	–	–	–	–	–	5.8^a^ (B)	8.7	2/9 (22%)	
Orbital muscles	–	4^a,b^ (B)	–	–	2.5^a^	–	–	–	–	3.3	2/9 (22%)	
White matter	2	1.8	2.9^a^	2.5^a^	2	2.8^a^	0.8	2.8^a^	3.4^a^	2.3	9/9 (100%)	
Gray Matter	1	0.9	3.4^a^	2.9^a^	1.3	1	0.8	2.6^a^	1.6	1.7	9/9 (100%)	
Skeletal Muscles	–	2.8^a^ (B)	–	–	–	–	–	2.2^a^	4.5^a^ (B)	3.2	3/9 (33%)	
Tongue	–	5.8^a^	2.3	–	3.3^a^	–	–	–	6.3^a^	4.4	4/9 (44%)	1/9 (11%)
Pleura	–	–	–	–	5.7^a^ (D)	–	3.7^a^ (D)	–	–	4.7	2/9 (22%)	

*D* diffuse, *B* bilateral, *F* focal

^a^Increased uptake above adjacent background was considered abnormal

^b^Positive MRI:

• bone marrow (patients #1, 4, 8): non-specific heterogeneous T1w marrow signal

• lungs (patient #2): basilar lung consolidation with moderate right and mild left pleural effusions

• stomach (patient #3): area of asymmetric wall thickening and lack of enhancement

• ocular muscles (patient #2): mild predominantly lateral rectus muscle thickening

## Discussion

Here, we argue for the role of whole-body amyloid imaging in assessing patients with suspected cardiac amyloidosis and possible systemic extent of disease. In this clinical scenario, PET/MRI offers the advantages of ^18^F-florbetaben’s specific binding to amyloid plaques with the structural information from MRI, which also has an established role in diagnosing cardiac amyloidosis. The whole-body evaluations of these nine patients illustrate the variable clinical manifestations of systemic amyloidosis, which classically tends to involve the heart, kidneys, lungs, or gastro-intestinal tract, but can also affect any other organ.

After the success of amyloid PET tracers in brain imaging, a marked interest emerged into the clinical applicability in cardiac amyloidosis. In a study by Law et al., diffusely increased myocardial ^18^F-florbetaben uptake was observed in all AL and ATTR patients, while none was observed in the hypertensive controls. Moreover, myocardial retention was an independent determinant of myocardial dysfunction in cardiac amyloidosis [5]. Of our five patients showing increased myocardial uptake, three had an echocardiogram with echo-texture suspicious for amyloid, one had MRI with extensive subendocardial delayed enhancement consistent with amyloidosis, and the last one had biopsy-confirmed cardiac involvement. Echocardiography of the remaining four patients revealed left ventricular hypertrophy but was otherwise nonspecific. This appears promising for the diagnostic dilemma of differentiating cardiac amyloidosis from other more common causes of myocardial thickening such as hypertensive heart disease.

Ezawa et al. recently showed that organ involvement on whole-body ^11^C-PiB imaging correlated well with clinical findings and histological amyloid deposition for the heart and stomach. Interestingly, however, abnormal tracer uptake was also observed in the spleen, lacrimal and salivary glands, brain, scalp, extraocular muscles, naso-oropharynx and nuchal muscles, most of which were asymptomatic. The majority of patients and even one healthy control subject showed asymptomatic tracer uptake in the lung and parotid gland [11].

There have been only two published cases of ^18^F-florbetaben for evaluation of amyloid systemic involvement. D’Estanque et al. presented an interesting image showing not only the suspected cardiac amyloidosis but also intense spleen and thyroid pathologic uptake and moderate salivary gland and kidney uptake [6]. Genovesi et al. reported a case with moderate diffuse uptake in the bone marrow and significant cortical brain uptake [7]. Skeletal ^18^F-florbetaben uptake of uncertain mechanism and significance had also been noted in a pilot cardiac imaging study [5].

In our case series, in addition to the physiologic uptake in the urinary tract and enterohepatic circulatory system related to clearance mechanisms and excretion pathways [12], abnormal ^18^F-florbetaben retention was observed in extracardiac sites of amyloid involvement, including the lungs and extraocular muscles. Indeterminate uptake was also seen in many of the sites described in the previous systemic amyloid imaging studies. Six patients demonstrated mostly diffuse ^18^F-florbetaben uptake in the stomach, while three patients each showed avid uptake in the salivary glands and seven diffuse uptake in the bone marrow, which are notably commonly involved organs [13–15]. Pathologic uptake revealed by systemic amyloid imaging offers a promising alternative to invasive biopsies, given the generally nonspecific radiographic appearance and variable clinical presentations, often asymptomatic at early stages of the disease.

It is noteworthy that the present study included only patients referred for known or suspected cardiac amyloidosis, representing a distinct population, as cardiac involvement is a well-known poor prognostic factor in itself. The pilot nature of the study lead to the small number of included patients. Nevertheless, whole body evaluations revealed a wide range of involvement in multiple organs. For more conclusive results, rigorous histopathologic correlation for each area of abnormal uptake is warranted in future larger studies.

## Conclusion

Whole-body ^18^F-florbetaben PET/MRI is promising for localization of systemic amyloid deposition. This technique may provide important structural and functional information regarding the organs involved by disease, with potential to guide biopsy and evaluate response to treatment.

## Additional file

Additional file 1: Details of the image acquisition. (DOCX 13 kb)

## References

[1] Falk RH, Dubrey SW (2010). Amyloid heart disease. Prog Cardiovasc Dis.

[2] Strosberg J, El-Haddad G, Wolin E, Hendifar A, Yao J, Chasen B (2017). Phase 3 trial of (177)Lu-Dotatate for Midgut neuroendocrine tumors. N Engl J Med.

[3] Dubrey SW, Hawkins PN, Falk RH (2011). Amyloid diseases of the heart: assessment, diagnosis, and referral. Heart.

[4] Villemagne VL, Ong K, Mulligan RS, Holl G, Pejoska S, Jones G (2011). Amyloid imaging with (18)F-florbetaben in Alzheimer disease and other dementias. J Nucl Med.

[5] Law WP, Wang WY, Moore PT, Mollee PN, Ng AC (2016). Cardiac amyloid imaging with 18F-Florbetaben PET: a pilot study. J Nucl Med.

[6] D'Estanque E, Chambert B, Moranne O, Kotzki PO, Boudousq V (2017). 18F-Florbetaben: a new tool for amyloidosis staging?. Clin Nucl Med.

[7] Genovesi D, Vergaro G, Emdin M, Giorgetti A, Marzullo P. PET-CT evaluation of amyloid systemic involvement with [(18)F]-florbetaben in patient with proved cardiac amyloidosis: a case report. J Nucl Cardiol. 2017. doi:10.1007/s12350-017-0856-5. 10.1007/s12350-017-0856-5 28326465

[8] Genovesi D, Vergaro G, Emdin M, Giorgetti A, Marzullo P (2017). PET-CT evaluation of amyloid systemic involvement with [(18)F]-florbetaben in patient with proved cardiac amyloidosis: a case report. J Nucl Cardiol.

[9] Joshi AD, Pontecorvo MJ, Adler L, Stabin MG, Skovronsky DM, Carpenter AP (2014). Radiation dosimetry of florbetapir F 18. EJNMMI Res.

[10] Wagner T, Page J, Burniston M, Skillen A, Ross JC, Manwani R, et al. Extracardiac (18)F-florbetapir imaging in patients with systemic amyloidosis: more than hearts and minds. Eur J Nucl Med Mol Imaging 2018;45:1129–1138. doi:10.1007/s00259-018-3995-2. 10.1007/s00259-018-3995-2 PMC5953997 29651545

[11] Ezawa N, Katoh N, Oguchi K, Yoshinaga T, Yazaki M, Sekijima Y. Visualization of multiple organ amyloid involvement in systemic amyloidosis using (11)C-PiB PET imaging. Eur J Nucl Med Mol Imaging. 2017; 10.1007/s00259-017-3814-1. 10.1007/s00259-017-3814-1 28891012

[12] O'Keefe GJ, Saunder TH, Ng S, Ackerman U, Tochon-Danguy HJ, Chan JG (2009). Radiation dosimetry of beta-amyloid tracers 11C-PiB and 18F-BAY94-9172. J Nucl Med.

[13] Lovat LB, Pepys MB, Hawkins PN (1997). Amyloid and the gut. Dig Dis.

[14] Suzuki T, Kusumoto S, Yamashita T, Masuda A, Kinoshita S, Yoshida T (2016). Labial salivary gland biopsy for diagnosing immunoglobulin light chain amyloidosis: a retrospective analysis. Ann Hematol.

[15] Swan N, Skinner M, O'Hara CJ (2003). Bone marrow core biopsy specimens in AL (primary) amyloidosis. A morphologic and immunohistochemical study of 100 cases. Am J Clin Pathol.

