# Supplementary material for: 18F-florbetaben whole-body PET/MRI for evaluation of systemic amyloid deposition
Source: EJNMMI Res. 2018 Jul 24;8:66. doi: 10.1186/s13550-018-0425-1 (PMC6057864; doi:10.1186/s13550-018-0425-1)
Supplement: Supplementary file 1 — Details of the image acquisition. (DOCX 13 kb) [file 13550_2018_425_MOESM1_ESM.docx]

**Additional file #1: details of the image acquisition**

Cardiac MRI sequences included breath-hold, ECG triggered balanced cine SSFP for assessment of ventricular size and function by a stack of short axis slices (slice thickness 8 mm, TR 3.0 ms, TE 1.5 ms, flip angle 50°, temporal resolution 35 ms; in-plane resolution 1.7x1.4 mm), and two-dimensional (2D) turbo inversion-recovery magnitude T2-weighted imaging in short axis for assessment of myocardial inflammation and edema (slice thickness 8 mm, TR 2000 ms, TE 44 ms, flip angle 180°, inversion time 180 ms, in-plane resolution 1.3x1.4 mm). Late gadolinium enhanced (LGE) imaging was performed 15 minutes following administration of 0.15 mmol/kg bodyweight of Multihance (Bracco Diagnostics Inc, Monroe Township, NJ) employing a 2D inversion recovery gradient-recalled echo sequence (IR GRE) in short-axis (slice thickness 8 mm, TR 6.5 ms, TE 1.5 ms; flip angle 20°; inversion times 220-360 ms; in-plane resolution 1.8x1.4 mm). Two-, three- and four-chamber LGE planes were also obtained. LGE imaging was not obtained in two patients due to documented history of renal insufficiency.

The whole-body PET acquisition was performed in 3D mode and 2 minutes/bed position (89 slices/bed) in 5-9 beds. An axial 2-point Dixon 3-dimensional T1-weighted spoiled gradient echo MR sequence (TR/TE1/TE2: 4.1/1.1/2.2 ms; FOV 50 x 37.5 cm; matrix 256 x 128; slice thickness/spacing: 5.2/2.6 mm; 120 images/slab; imaging time 18 sec) was acquired at each table position and used to generate attenuation correction (AC) maps and for anatomic registration of the PET results. PET images were reconstructed using ordered subset expectation maximization (OSEM) protocol with 2 iterations and 28 subsets. TOF reconstructed images assumed a Gaussian kernel of 400 ps width. The Dixon MRI sequence and the PET acquisition started at the same table position and times, thus ensuring optimal temporal and regional correspondence between MRI and PET data. For AC, the images were segmented into different tissue types with an anatomy-aware algorithm, and were co-registered to a CT atlas in the head region. T1-weighted (T1w) axial 2-point Dixon 3D spoiled gradient echo were acquired at each station, as recommended by the vendor (LAVA-flex; TR/TE1/TE2: 4.9/1.3/2.5 ms; flip-angle 15**°**; bandwidth: 142.86kHz; FOV: 44 cm; matrix: 320 x 224; slice thickness 3.4-mm; ARC acceleration factor: 2x2; imaging time 21-55 minutes).
